# Hyperpolarized [1-^13^C]pyruvate magnetic resonance spectroscopic imaging identifies elevated lactate in epileptic tissue

**DOI:** 10.1093/braincomms/fcaf357

**Published:** 2025-09-13

**Authors:** Aditya Jhajharia, Mitchell Moyer, Jemima Olu-Owotade, Darrian McAfee, Abubakr Eldirdiri, Joshua Rogers, Minjie Zhu, Ujwal Boddeti, Riccardo Serra, J Marc Simard, Volodymyr Gerzanich, Muznabanu Bachani, Dirk Mayer, Alexander Ksendzovsky

**Affiliations:** Department of Diagnostic Radiology and Nuclear Medicine, University of Maryland School of Medicine, Baltimore, MD 21201, USA; Department of Neurosurgery, University of Maryland School of Medicine, Baltimore, MD 21201, USA; Department of Neurosurgery, University of Maryland School of Medicine, Baltimore, MD 21201, USA; Department of Neurosurgery, University of Maryland School of Medicine, Baltimore, MD 21201, USA; Department of Diagnostic Radiology and Nuclear Medicine, University of Maryland School of Medicine, Baltimore, MD 21201, USA; Department of Diagnostic Radiology and Nuclear Medicine, University of Maryland School of Medicine, Baltimore, MD 21201, USA; Department of Diagnostic Radiology and Nuclear Medicine, University of Maryland School of Medicine, Baltimore, MD 21201, USA; Department of Neurosurgery, University of Maryland School of Medicine, Baltimore, MD 21201, USA; Department of Neurosurgery, University of Maryland School of Medicine, Baltimore, MD 21201, USA; Department of Neurosurgery, University of Maryland School of Medicine, Baltimore, MD 21201, USA; Department of Pathology, University of Maryland School of Medicine, Baltimore, MD 21201, USA; Department of Physiology, University of Maryland School of Medicine, Baltimore, MD 21201, USA; Department of Neurosurgery, University of Maryland School of Medicine, Baltimore, MD 21201, USA; Department of Neurosurgery, University of Maryland School of Medicine, Baltimore, MD 21201, USA; Department of Diagnostic Radiology and Nuclear Medicine, University of Maryland School of Medicine, Baltimore, MD 21201, USA; Fischell Department of Bioengineering, University of Maryland, College Park, MD 21201, USA; Department of Neurosurgery, University of Maryland School of Medicine, Baltimore, MD 21201, USA

**Keywords:** hyperpolarized ^13^C, magnetic resonance imaging, spectroscopy, epilepsy, lactate

## Abstract

Thirty percent of epilepsy patients have seizures despite best medical therapy. While epilepsy surgery has emerged as a promising treatment option for these patients, surgical outcomes vary considerably between patients and have not significantly improved over the years. These stagnant outcomes can be attributed to poor seizure onset zone and epileptic network localization with currently available tools. Lactate production is a well-known consequence of metabolic reprogramming and biomarker in epilepsy. Detection of lactate elevations using conventional magnetic resonance spectroscopy has been extensively studied as an effective tool to non-invasively detect epileptic brain tissue. However, this method suffers from poor spatial resolution, which limits its clinical utility in presurgical resection mapping. In this study, we explore the utility of a recently developed approach, magnetic resonance spectroscopy and spectroscopic imaging of hyperpolarized [1-^13^C]pyruvate, to identify epileptic tissues via detection of increased lactate production. We found that this approach accurately identifies elevated lactate production in an *in vitro* model of chronic hyperactivity and in the gold standard mouse epilepsy model, pentylenetetrazol kindling. These data suggest that magnetic resonance spectroscopic imaging of hyperpolarized [1-^13^C]pyruvate has the potential to effectively and non-invasively map epileptic foci and should be further explored as a clinical tool to guide epilepsy resection surgery by identifying epileptic tissue in patients.

## Teaser

Jhajharia *et al*. investigated whether hyperpolarized ^13^C magnetic resonance spectroscopy (HP ^13^C MRS) effectively identifies epileptic tissues defined by elevated lactate production, a well-established biomarker. Authors observed HP ^13^C MRS effectively differentiates epileptic from non-epileptic conditions in vitro and in vivo, supporting its clinical role for presurgical planning for tissue resection.

## Graphical Abstract

**Graphical Abstract:**
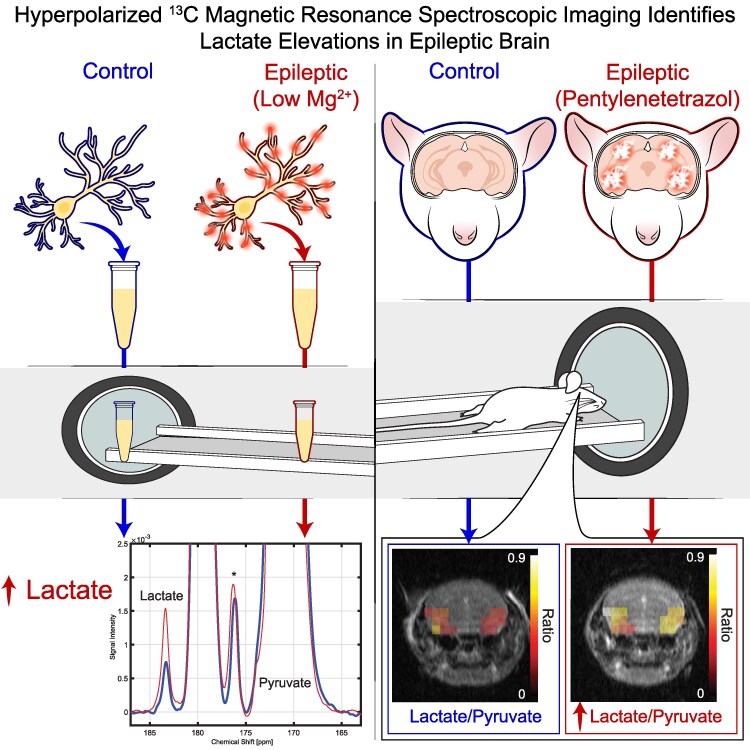

## Introduction

Approximately 30% of patients with epilepsy continue to experience seizures despite best medical management.^1^ This persistence of seizures, often accompanied by the use of multiple medications,^2^ significantly impacts the patient’s quality of life.^3^ In these patients, epilepsy surgery is a well-established treatment. However, despite the advancement of preoperative epilepsy assessments that integrate newer non-invasive techniques and imaging methods, the improvements in surgical outcomes remain marginal.^4,5^ The lack of progress in these outcomes has been attributed to challenges in precisely identifying the seizure onset zone and the epileptic network using existing tools.^4,5^ Hence, developing new non-invasive methods based on detailed molecular insights, such as metabolic biomarkers, holds the promise of accurately locating the seizure onset zone and epileptic network, potentially enhancing surgical outcomes.

Epileptic tissue is characterized by hyperexcitability that makes neuronal populations susceptible to high-frequency synchronous firing underlying seizure events.^6^ Increased excitability in epileptic neurons is associated with alterations in cellular processes such as synaptic plasticity, neuroinflammation, gene regulation and metabolism.^7,8^ Regarding metabolism, chronic hyperactivation in epileptic brain leads to a shift in glucose metabolism from oxidative phosphorylation to glycolysis.^8,9^ Thus, elevated glycolytic metabolism may be a useful biomarker to differentiate normal from epileptic brain tissues.

Utilizing metabolic imaging for presurgical mapping of epileptic tissues represents a promising avenue to improve upon the current standard-of-care seizure localization methods.^10-12^ In particular, elevation in brain lactate (Lac) production is a promising biomarker of glycolytic metabolism, as elevated Lac is classically associated with acute seizures and may remain persistently elevated interictally.^13-19^ Recent work by us and others has suggested that human epileptic tissues demonstrate increased interictal lactate dehydrogenase A (LDHA) protein expression compared to non-epileptic within-patient control samples, and that LDHA expression and Lac conversion are increased in rodent and *in vitro* chronic epilepsy models.^15,20,21^ Thus, persistent interictal Lac may serve as a useful biomarker for *in situ* differentiation of epileptic tissues from healthy tissue during presurgical planning for resection surgery. In fact, serum, cerebrospinal fluid (CSF), and brain parenchymal Lac elevations have all been studied and proposed as useful biomarkers for identifying epilepsy patients and epileptic brain tissue.^20,22-24^

Conventional magnetic resonance spectroscopy (MRS) has been extensively studied preclinically and in clinical trials for its ability to non-invasively map Lac elevations and aid in resection surgeries.^25-31^ However, the inherently small millimolar concentration of most metabolites in human tissues leads to a relatively low signal-to-noise ratio, limiting its use. These small concentrations hamper the achievable spatial resolution within a clinically acceptable scan time and creating challenges in discerning overlapping peaks at clinical field strengths. Addressing these challenges is critical for ensuring that this technology is useful in preoperative planning for epilepsy surgery.^32^ Given its properties, ^13^C MRS of thermally polarized, ^13^C enriched tracers may address these challenges. ^13^C MRS is able to track metabolic pathways, such as the tricarboxylic acid cycle, using ^13^C tracers like labelled glucose in both healthy and pathological conditions.^33-35^ Despite ^13^C enrichment for the injected compound, the signal-to-noise ratio remains low due to the low gyromagnetic ratio and long T1 values, restricting the technique's achievable spatial and temporal resolutions in typical 3T clinical settings. Hyperpolarized (HP) ^13^C magnetic resonance imaging (MRI) overcomes these problems by increasing MR signals up to five orders of magnitude.^36^ As such, ^13^C MRS using HP tracers offers a rapid, non-invasive, and sensitive means of investigating metabolic processes. Using [1-^13^C]pyruvate (Pyr) as the substrate, this approach permits real-time detection of Pyr’s downstream metabolic products, including Lac and bicarbonate, shedding light on glycolysis and oxidative phosphorylation pathways and potentially assisting with identifying epileptic tissue. HP ^13^C magnetic resonance spectroscopic imaging (MRSI) is a cutting-edge imaging technology that takes advantage of the above properties and enables the spatial and temporal localization of metabolic by-products, such as Lac, for clinical use in conditions like glioma.^37-39^ Therefore, as the metabolic underpinnings of epilepsy are further investigated, MRSI of HP [1-^13^C]Pyr could play a significant role in seizure onset zone identification.

Our study aims to make progress using MRSI of HP [1-^13^C]Pyr to identify epileptic brain tissue. Based on prior MRS studies and HP ^13^C MRSI studies done in glioma, we hypothesized that MRS/I of HP [1-^13^C]Pyr would successfully detect Lac elevations in epileptic conditions *in vitro* using a chronic low Mg^2+^ hyperactivation model and *in vivo* using pentylenetetrazol (PTZ) kindling. These models were chosen based on our prior findings demonstrating LDHA upregulation and/or increased Lac conversion.^15^ Here, we found that MRS/I of HP [1-^13^C]Pyr detects increased Pyr-to-Lac conversion in preclinical models, providing evidence for the potential clinical applicability of HP ^13^C MRSI in mapping of the seizure onset zone and epileptic network in epilepsy.

## Materials and methods

### Ethics statement

The collection of animal tissue samples for this study was approved as part of the study protocol. This animal study was conducted in accordance with ARRIVE guidelines and was approved by the Institutional Animal Care and Use Committee (IACUC) at the University of Maryland, USA.

### Subjects

For *in vitro* experiments, embryonic day 18 pregnant female Sprague–Dawley rats (*n*  *=* 3 litters) were obtained from Charles River Laboratories (Wilmington, MA, USA). After birth, primary mixed cortical cultures were generated from postnatal day 2 (P2) pups as described below. For *in vivo* experiments, 6-week-old female C57/Bl6N mice (*n*  *=* 18 total mice) were obtained from Charles River Laboratories. Animals were randomly assigned groups, and the sample size was based on prior HP ^13^C MRSI studies in rodent brain (∼5–10 animals).^40-42^

### Rat mixed cortical model

#### Primary mixed cortical culture generation, seeding and maintenance

Rat cortical cultures were generated as described previously.^15^ New-born rat pups were anesthetized with 5% isoflurane with 0.8 L/min O_2_, brains were extracted, then cortices were dissected in a modified Puck’s dissociation medium [100 mL 20X D1 (8% NaCl, 0.045% KCl, 0.03% Na_2_HPO_4_⸱7H_2_O, 0.0012% KH_2_PO_4_ in deionized water), 100 mL glucose/sucrose solution (6% anhydrous glucose + 14.8% sucrose in deionized water), 50 mM HEPES buffer, pH to 7.4, osm to 320–330] maintained at 4°C. After dissection, cortices were dissociated in Puck’s/papain solution [1.5 mM CaCl_2_, 0.5 mM EDTA, 0.75% papain (Worthington Biochemical Corporation, Lakewood, NJ) and 8.25 nM cysteine in D1 medium], then cells were resuspended in seeding media (5% FBS, 1X B-27 supplement, 2X antibiotic/antimycotic mix, 5 mM HEPES, and 1.2 mM L-glutamine in Neurobasal medium, pH 7.4). Cells were seeded (200 000 cells/well) into either standard (*n* = 2) (Grenier Bio-One, Frickenhausen, Germany) or multi-electrode (MEA) (*n* = 3) (Axion Biosystems, Atlanta, GA, M768-tMEA-96W) 96-well plates precoated with Poly-D-Lysine (1 mg/mL diluted in borate buffer pH 8.4). Twenty-four hours after seeding, a full media change from seeding to maintenance media (5% FBS, 1X B-27 supplement, 1X antibiotic/antimycotic mix, 5 mM HEPES and 1.2 mM L-glutamine in Neurobasal medium, pH 7.4) was performed. Cells were subsequently maintained with media changes every 4 days until the beginning of the treatment period on day *in vitro* (DIV) 17.

#### Low magnesium treatments

We used a low Mg^2+^  *in vitro* model of neuronal activation to test the ability of HP MRS to identify elevated Lac in a model system in which our previous work demonstrated LDHA upregulation and increased Lac conversion.^15^ For low Mg^2+^ treatment, mixed cortical cultures were changed for 2 h from maintenance media to Mg^2+^ depleted aCSF (Low Mg^2+^: 10 mM HEPES solution, 2.5 mM KCl, 2 mM CaCl_2_, 2 μM glycine, 10 mM glucose, 145 mM NaCl and 0 mM MgCl_2_ in deionized water, pH 7.3). After 2 h, treatment aCSF was exchanged for maintenance media. To prevent cell loss, control cells were not treated with aCSF, instead undergoing maintenance media changes every 3 days, increased from 4 days to support increased neuronal activity.^15^ MEA-seeded cultures were recorded for 5 min before and after treatments to obtain baseline and post-treatment neuron population activity measurements. Baseline activity was monitored from DIV10 to DIV17, and only culture sets demonstrating sufficient and stable firing (≥6 Hz mean firing rate) by DIV17 were used. Treatments began on DIV17, when neuronal firing stabilizes, and occurred for 2 h daily for 10 days to induce chronic seizure-like hyperactivity. Each 96-well plate contains both conditions (48 wells per group). HP ^13^C MRS scans were then performed 24 h after the last control/low Mg^2+^ treatment, and cell supernatants were collected for Lac assay measurements prior to scanning.

#### MEA analysis

Cortical rat cultures on MEA plates were recorded using a Maestro Pro MEA system (Axion BioSystems, Atlanta, GA). Each well contains eight electrodes that record extracellular voltage with a sampling rate of 12.5 kHz. We used the Neural Metrics Tool (Axion BioSystems, Atlanta, GA) for neuronal action potential (spike), defined as when the recorded trace exceeded a threshold of ±6 standard deviations from the baseline signal, and burst, occurrences of five spikes on a single electrode with a maximal inter-spike interval of 100 ms identification, and for subsequent analyses. During every 5-min pretreatment and post-treatment recording, we computed the rate of neuronal bursts in every electrode in each well, then averaged electrode responses per well. To account for cell death or scarcity in an electrode’s vicinity, only wells with eight electrodes demonstrating a minimum spike rate of 5 spikes/minute, and a mean baseline firing rate of at least 6 Hz were considered in neural activity analysis. In each 5-min recording, neuron population burst frequency for each well was computed and averaged within each treatment group. This generated pretreatment and post-treatment burst frequency values for each treatment for each day. From these values, to assess the effect of either control or Low Mg^2+^ aCSF on neuron activity, we calculated the burst frequency ratio for each group within each day defined as the post-treatment/pre-treatment burst frequency. Three of the five treatment plates (each plate containing 48 wells of each condition) used in HP ^13^C MRS experiments were measured by MEA, while the other two were scanned from standard 96-well plates without MEA measurements.

### Pentylenetetrazol kindling

To model epilepsy, PTZ kindling model was induced in 6-week old female mice (*n* = 9) by intraperitoneal administration of sub-convulsive PTZ doses (35 mg/kg in 1X dPBS) (Sigma-Aldrich, Miamisburg, OH) every other day for 20 days as previously described.^15,43^ Mice were given dPBS (vehicle) on the same schedule as PTZ mice as a sham control [*n* = 9 initially, one control mouse’s results were excluded as a statistical outlier (>2 SD above mean for Lac assay measurements)]. Previous studies demonstrated that this model leads to Lac elevation in the hippocampus and cortex of mice.^15,44,45^ Behavioural seizure responses were scored 30 min after each injection using Racine Scale.^46^ The following classifications were used: 0: no seizure response, 1: behavioural arrest or slowing, 2: head nodding associated with facial clonus, 3: partial limb clonus with or without tail stiffening, 4: clonic seizure with rearing and loss of posture, 5: generalized tonic-clonic seizure with wild running and/or jumping, 6: death. Scoring was independently performed by two investigators unblinded to condition, however, no seizure phenotypes were exhibited by sham animals. HP ^13^C MRSI scans were performed 48 h after the last PTZ dose (i.e. 48 h after the last seizure, which we considered interictal).

### Lactate assay measurements

For *in vitro* measurements, the supernatant was collected immediately before cell resuspension for to assess extracellular Lac concentrations in aCSF-treated cells. For *in vivo* measurements, HP ^13^C MRSI scanned mice were euthanized by CO_2_ inhalation after MRSI and transcardially perfused with ice cold 0.9% normal saline. Brains were harvested, the cerebellum was removed, and one hemisphere was homogenized in 1 mL of cold 1X PBS. Regional dissections for temporal cortex and hippocampus were intentionally not performed to reduce the time from sacrifice to homogenization. After homogenization, samples were centrifuged at 1000 rpm for 10 min, and the resulting supernatant was removed for Lac analysis. A fluorometric Lac assay kit (Cell Biolabs, Inc. MET-5013) was used on collected *in vitro* and *in vivo* supernatant and homogenized brain, respectively, as recommended. Fifty microlitres of the lysate sample was incubated with 50 µL of the Reaction Mix in a well for 30 min at 37°C protected from light. The plate was read with a colorimetric microplate reader (Tecan, Männedorf, Switzerland). Obtained excitation (565 nm) emission (595 nm) values were used to calculate the Lac concentration of the sample based on a L-lactate standard reagent curve.

### HP ^13^C MRS/I measurements

#### Hyperpolarization of [1-^13^C]pyruvate

A multi-sample cryogenic dissolution dynamic nuclear polarization system (SPINLab, GE Healthcare, Niskayuna, NY, USA) operating at 5 Tesla (T) and cryogenic temperatures near 1 K was used to generate the HP solution as previously described.^47^ The polarized sample consisted of approximately 100 µL of neat [1-^13^C]pyruvic acid (Sigma–Aldrich, Miamisburg, OH) with 15 mM trityl radical. The pyruvic acid sample was polarized using microwave irradiation at 139.8 GHz for at least 2.5 h. After dissolution with an aqueous solution of 0.1 g/L ethylenediaminetetraacetic acid disodium salt dihydrate (EDTA-Na_2_) heated to 130°C at 4.5 bar, the HP Pyr solution was neutralized with a buffer solution consisting of 0.72 M NaOH, 0.4 M Trizma base, and 0.1 g/L EDTA-Na_2_ resulting in a near physiological pH of 6.40 ± 0.34.

#### In vitro HP ^13^C MRS data acquisition and analysis

HP ^13^C MRS studies (*n* = 5 treatment plates) were conducted using a clinical 3 T MRI scanner with non-proton option (Discovery 750w, GE Healthcare, Waukesha, WI, USA). Twenty-four hours after the last aCSF treatment, low Mg^2+^ and control cells (48 wells of each group per plate) were detached with 0.05% trypsin, combined into a single sample within each treatment group, centrifuged at 1000 rpm for 5 min, then resuspended in 2 mL of media to create a cell suspension containing approximately 1 × 10^7^ cells for each sample. This suspension was then transferred to a 30 mL syringe, and a custom-built, single-loop ^13^C radio frequency (RF) coil (inner diameter = ∼40 mm) was placed near the 2 mL horizontal mark of the syringe positioned vertically at the centre of the magnet for data acquisition. ^13^C-MRS signals for each set of cells were then obtained after administering a 2 mL bolus injection in approximately 8 s of ∼14 mM HP [1-^13^C]Pyr solution through a thin injection line ending at the bottom of the 30 mL syringe to ensure uniform mixing of cells and HP solution.^48,49^

MR measurements were performed with ^13^C RF coil used for both RF excitation and signal reception. A non-selective RF excitation pulse (32-µs duration) with a nominal flip angle of 5.6° was used to preserve magnetization for subsequent acquisitions. Both ^13^C frequency and RF power were calibrated using an 8-M ^13^C-urea phantom placed near the centre of the surface coil that was removed prior to adding the cell suspension. The spectra were acquired with a repetition time (TR) of 3 s for 80 scans, utilizing a spectral width of 10 kHz and 4096 spectral points. The total acquisition time for the spectra was 4 min. Data were processed using custom code (MATLAB, MathWorks Inc.). A 10-Hz Gaussian filter and zero-filling by a factor of 2 in the time dimension were applied to the acquired data sets. Subsequently, a fast Fourier transform (FFT) was performed, and metabolite peaks were integrated in absorption mode following zero-order phase correction to quantify time curves of the metabolites. Pyr and Lac were quantified by integrating the respective peak in the spectrum that was averaged over the first 60 time points (180 s). The Lac-to-Pyr ratio was normalized to total cell count taken after each scan to account for variations between groups in live cell counts during experiments then compared in control versus low Mg^2+^ groups.

#### In vivo HP ^13^C MRSI data acquisition and analysis

*In vivo* MRSI of HP [1-^13^C]Pyr experiments were conducted using a preclinical 3 T MRI scanner (Biospec 3T, Bruker, Ettlingen, Germany). A custom-built single-loop surface coil (inner diameter: 40 mm) used for RF excitation and signal reception. The animals were scanned in the prone position with the surface coil positioned above the animal’s head cantered around the brain. The animal together with the ^13^C surface coil was inserted into a quadrature ^1^H volume coil (inner diameter: 72 mm) for proton data acquisition. The surface coil’s position was confirmed using a fiducial marker at the centre of the coil along the *z*-axis visible on the ^1^H GRE localizer images. Animal body temperature was maintained at ∼37°C using a warm water blanket underneath the animal regulated by temperature feedback from a rectal temperature probe. Animals were anesthetized throughout the imaging session using continuous isoflurane (1–2% in 1 L/min O_2_), and respiration was measured using a pressure sensor placed underneath the animal (model 1030, Small Animal Instruments, Inc., Stony Brook, NY, USA). The anaesthesia was tightly controlled based on the respiration rate and kept at a similar level for all scanning sessions as it is a potent cerebral vasodilator and variations can affect the product-to-substrate ratios in brain applications of HP ^13^C MRSI.^50^

After acquiring ^1^H GRE localizer images along all three principal axes, shimming, and ^13^C RF power and frequency calibration, a fast dynamic spiral chemical shift imaging (spCSI)^51^ scan with a multiband spectral-special RF pulse^52^ was applied for data acquisition. The ^13^C transmit power and flip angle were calibrated using an 8 M solution of ^13^C labelled urea syringe placed on top of the animal’s head. The ^13^C frequency was calculated from the brain water ^1^H frequency measured with point-resolved spectroscopy using a predetermined scaling factor that depends on the target chemical shift.^53^ The ^13^C RF power calibration was performed by minimizing the signal from the urea phantom using a 180° reference pulse. Animals were injected with ∼114 mM of HP Pyr (10 µL/g body weight) through a tail vein catheter 4–5 s after starting the spCSI scan. A multiband RF pulse permitted different excitation flip angles for substrate and product resonances to preserve the longitudinal magnetizations of the substrate for dynamic imaging and to increase the signal-to-noise ratio of metabolic products. We applied a 1° flip angle on Pyr and 4° on Lac, corresponding to effective flip angles per time point of 6° and 22°, respectively. The other parameters of the spCSI sequence were: FOV = 32 × 32 × 32 mm^3^, 8 phase encoding steps in slice direction for a nominal resolution of 2 × 2 × 4 mm^3^, 4 spatial interleaves, 56 spiral gradient echoes, TE = 4.2 ms, TR = 118 ms, spectral width = 531 Hz. A total of 20 data sets were acquired for an 80-s scan at 4 s temporal resolution.

T2-weighted (T2w) proton images were acquired before or after the ^13^C acquisition using a 3D TurboRARE sequence for anatomical reference. Parameters for the T2w sequence were as follows: TE = 69 ms, TR = 1800 ms, two averages, with 32 × 32 × 32 mm^3^ FOV. The image size was 256 × 128 × 32 with 0.125 × 0.250 × 1 mm^3^ resolution.

As some studies suggest that PTZ kindling can cause elevated blood–brain barrier (BBB) disruption,^54,55^ and transport across the BBB is a rate-limiting step in the conversion of HP Pyr to Lac,^56^ we acquired T1-weighted (T1w) proton images before and approximately 2 min after intravenous (IV) injection of 100 µL of a 1:2 mixture of a macrocyclic gadolinium-based contrast agent (Gadavist, Bayer HealthCare Pharmaceuticals, Whippany, NJ) and saline to measure BBB permeability for all mice.^47^ Parameters for the 2D T1w MRI were: TE = 12 ms, TR = 400 ms, eight averages, FOV = 40 × 40 mm^2^ with 128 × 128 image matrix and nine slices with 1 mm slice thickness. The T1w scans were always performed within the same MRI session approximately 5–15 min after ^13^C imaging to avoid any ^13^C signal attenuation due to the presence of the T1-shortening contrast agent.

To ensure uniformity and consistency among all animals, for each animal the brain was manually co-registered based on the ^1^H MRI to a standardized position with a rigid transformation consisting of translation along the three principal axes and a rotation around the *z*-axis. The transformation parameters obtained from ^1^H MRI were applied to the 3D ^13^C spCSI data in the *k*-space domain followed by reconstruction using a custom-build MATLAB code similar to that previously described.^57^ In brief, the data were apodized in the spatial *k*-space dimensions with a generalized Hanning window (alpha = 0.66) and zero-filled by a factor of 2 in the in-plane dimensions. Then, the echo dimension was processed with combined Gaussian/Lorentzian (20 Hz/−5 Hz) apodization and zero-filling by a factor of 4 followed by FFT to convert it to the spectral domain. A frequency-dependent linear phase correction was applied along the readout direction followed by inverse FFT along the slice dimension. The remaining *k*-space dimensions were converted into spatial dimensions using an inverse non-uniform FFT.^58^ Metabolite maps for Pyr and Lac were calculated by peak integration after correcting the data for the respective effective excitation flip angle and phasing the spectrum in each voxel to produce an absorption-mode signal and integrating the resulting peak with an integration width of 22 Hz.

Regions of interest (ROIs) in the ventral hippocampus and temporal cortices were bilaterally drawn manually using proton T2w axial images while avoiding regions of parenchyma affected by high Pyr signal from vascular structures superior and inferior of the brain due to partial volume effects. After down-sampling to the resolution of the metabolic maps, the identical ROI was applied to all mice to maintain consistency. The Lac-to-Pyr ratio in the ROIs was calculated as the ratio of the mean Lac and Pyr in the ROIs measured in the sum of all 16 time points.

### Statistical analysis

A one-tailed paired *t*-test was used to detect increases in the Lac/Pyr ratio and Lac assay measurements between control and low Mg^2+^ cell treatment groups, while one-tailed unpaired *t*-test was employed for control versus PTZ mice. Note that different RF coils with slightly different sensitivity profiles were used for these *in vitro* experiments leading to variations in signal intensities. However, as the groups were always measured in pairs of one each control and treatment sample per plate, the paired *t*-test is appropriate. The one-tailed *t*-test was employed, as the objective of these studies was to demonstrate that HP ^13^C MRSI can differentiate epileptic from non-epileptic tissue by detecting elevations in Lac production, which has been extensively shown in prior literature to occur.^13-19^ Thus, the negative tail of these analyses (i.e. detecting a significant decrease in Lac) is not relevant. A two-tailed unpaired *t*-test was used to compare the pregadolinium, post-gadolinium, or change in gadolinium signals in PTZ versus sham animals. A correlation analysis was performed between HP ^13^C MRSI Lac/Pyr ratio measurements and Lac assay measurements from brain ROIs. All values are reported as mean ± standard error of the mean (SEM). We designated the level of significance for all statistical tests as *P* < 0.05. Full statistical details, as well as sample sizes and their representations, are listed in figure legends. We used GraphPad Prism (version 9.3.1, San Diego, CA, USA) for all statistical analyses.

## Results

### HP ^13^C MRS successfully detects elevations in lactate production associated with neuronal hyperactivity after low Mg^2±^ treatment

**Figure 1:**
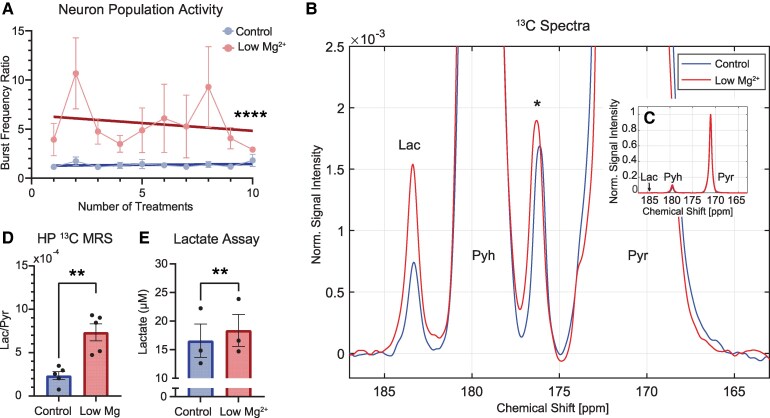
**MRS of HP [1-^13^C]Pyr can be used to detect elevated Lac induced by chronic neuron hyperactivation. (A**) Daily neuron activity measurements demonstrate increased population bursting (post-treatment/pretreatment burst frequency ratio) in response to low Mg^2+^ compared to control treatments (*P* < 0.0001, *n* = 3 MEA 96-well plates with 48 wells of each condition, *F* = 26.55, simple linear regression). (**B**) Representative time-averaged ^13^C spectra produced by low Mg^2+^ and control treated cells (zoomed in). Each spectrum was normalized to the Pyr peak. The spectra demonstrate elevated Lac peaks relative to the Pyr signal in low Mg^2+^ treated cells. The * marks a contamination present in the HP Pyr solution. (**C**) Representative time-averaged ^13^C spectra (zoomed out insert). (**D**) Lac/Pyr ratios in low Mg^2+^ treated cells detected via HP ^13^C MRS were significantly increased (*P* = 0.0081, *n* = 5 96-well plates with 48 wells of each condition, *t* = 4.002, one-tailed paired *t*-test) compared to control cells. (**E**) Extracellular Lac assay analysis, shown in measured concentrations (*P* = 0.0026, *n* = 3 96-well plates with 48 wells of each condition, *t* = 13.87, one-tailed paired *t*-test) detected significantly increased Lac in low Mg^2+^ treated conditions. *P < 0.05, **P < 0.01, one-tailed paired *t*-test; ****P < 0.0001. Abbreviations: Lac, lactate; Pyr, pyruvate; Pyh, pyruvate hydrate.

To assess whether elevations in Lac production from seizure activity could be detected with HP ^13^C MRS, Lac concentration changes were first characterized in an *in vitro* model of chronic neuronal hyperactivation associated with increased LDHA expression, the enzyme responsible for Lac production.^15^ As previously described, daily low Mg^2+^ treatment of mixed cortical cells for 10 days increased (*P* < 0.0001) daily neuronal bursting (Fig. 1A). On treatment day 11, HP ^13^C MRS and a Lac assay were independently used to compare Lac in control versus low Mg^2+^ treated conditions. In both control and treated conditions, [1-^13^C]Lac produced from [1-^13^C]Pyr via LDH was detected at 183 ppm (Fig. 1B and C). Pyr hydrate (Pyh) at 179 ppm was in dynamic equilibrium with Pyr and itself not metabolically active. Each spectrum was averaged over the first 60 time points and normalized to the [1-^13^C]Pyr peak at 171 ppm. HP ^13^C MRS detected a nearly 3-fold increase (*P* = 0.008) in mean Lac/Pyr ratio in low Mg^2+^-treated cells compared to control when normalized to live cell counts (Fig. 1D), which represents an increase in Lac conversion from Pyr in low Mg^2+^ cells. Additionally, increased Lac concentrations in low Mg^2+^ supernatants compared to controls (*P* = 0.02) were detected by Lac assay (Fig. 1E), further validating the HP ^13^C MRS result. Together, these results suggest that HP ^13^C MRS can effectively detect true biological increases in Lac production induced by chronic neuronal hyperactivation.

### MRSI of HP [1-^13^C]Pyr successfully detects elevated lactate after chronic seizures in temporal cortex and hippocampus of PTZ-kindled mice

**Figure 2:**
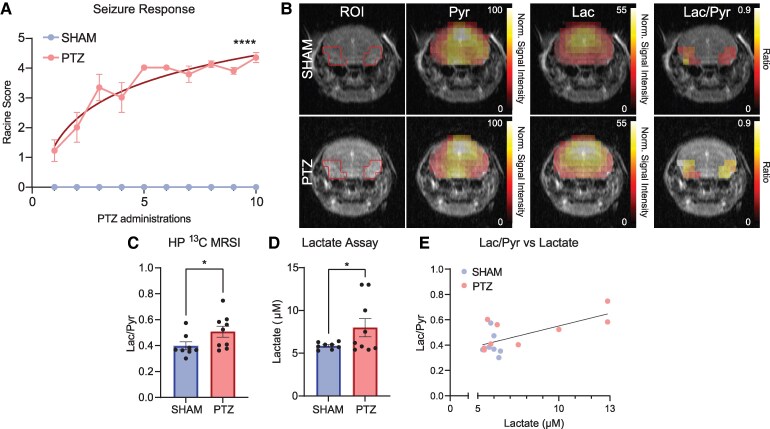
**MRSI of HP [1-^13^C]Pyr detects elevated lac in epileptic mice in brain regions associated with chronic seizures.** (**A**) Behavioural data from PTZ kindling induced prior to scanning mice demonstrate gradually increasing seizure response (Racine) score (*P* < 0.0001, *n* = 8 SHAM, *n* = 9 PTZ mice, *F* = 555.4, nonlinear regression with least squares fit). (**B**) MRI image with superimposed boundaries highlighting the ROIs (temporal cortex, ventral hippocampus) used for HP ^13^C MRSI measurements. Metabolic images (FOV = 2 × 32 mm^2^, 4-mm slice) of Pyr, Lac and Lac/Pyr acquired from SHAM (top) and PTZ (bottom) animals superimposed onto the T2w proton MRI. Metabolite intensities are normalized to the maximum in the respective Pyr map, and Lac/Pyr images show the magnitude of the Lac/Pyr ratios. PTZ animals show higher Lac/Pyr in temporal cortex and hippocampus compared to SHAM animals. (**C**) HP ^13^C MRSI comparison between PTZ and control mice demonstrated a significant increase in Lac/Pyr ratios within ROIs of epileptic mice (*P* = 0.0307, *n* = 8 SHAM, *n* = 9 PTZ mice, *t* = 2.021, one-tailed unpaired *t*-test). (**D**) Lac concentration measurements from brain homogenates of scanned mice demonstrate increased Lac in PTZ mice compared to controls, shown as measured Lac concentrations (*P* = 0.041, *n* = 8 SHAM, *n* = 9 PTZ mice, *t* = 1.864, one-tailed unpaired *t*-test). (**E**) Correlation analysis comparing MRSI of HP [1-^13^C]Pyr-defined Lac/Pyr ratios to brain lactate concentrations detected by Lac assay shows a moderate correlation between Lac signals measured independently in the same samples by either HP ^13^C MRSI or Lac assay (*r* = 0.6559, *R*^2^ = 0.4302, *P* = 0.0042, *n* = 17 mice). This validates MRSI of HP [1-^13^C]Pyr as a viable tool to effectively detect true biological increases in Lac conversion in epileptic brain tissues. Representative spectra from 3 mice per group are available in Supplementary Fig. 1*. Abbreviations*: HP ^13^C MRSI, hyperpolarized ^13^C magnetic resonance spectroscopic imaging; Lac, lactate; Pyr, pyruvate; PTZ, pentylenetetrazol.

To determine whether elevated Lac from chronically induced seizures can be detected using MRSI of HP [1-^13^C]Pyr *in vivo*, we assessed Lac measurements in brain of sham versus PTZ-kindled mice 48 h after the last PTZ-induced seizure. As is characteristic of PTZ kindling,^43^ behavioural seizure severity (Racine) scores progressively increased (*P* < 0.0001) over the course of PTZ kindling induction until chronic seizures were seen in PTZ-kindled animals (Fig. 2A). No seizures were witnessed in sham animals. Lac/Pyr signals acquired after injection of HP [1-^13^C]Pyr were measured within predefined ROIs in the ventral hippocampus and temporal cortices of animals, in accordance with prior literature suggesting the involvement of these regions in PTZ-induced seizures (Fig. 2B, Supplementary Fig. 1).^59-61^  ^13^C spectra averaged over the ROIs from three representative animals in each of the two groups are shown in Supplementary Fig. 1. Significantly elevated Lac/Pyr ratios (*P* = 0.0307) were detected using HP ^13^C MRSI within the temporal cortical and hippocampal ROIs of PTZ kindled animals compared to sham controls (Fig. 2B and C). No difference in concentration of the injected Pyr solution between the two groups was found (Control: 114.24 ± 2.22 mM; PTZ: 112.70 ± 3.63 mM; *n* = 8 sham, *n* = 9 PTZ mice, *P* = 0.3166, two-tailed unpaired *t*-test). A Lac assay analysis of brain homogenates after the HP ^13^C MRSI procedure confirmed high Lac in PTZ compared to sham controls (*P* = 0.041) (Fig. 2D).

**Figure 3:**
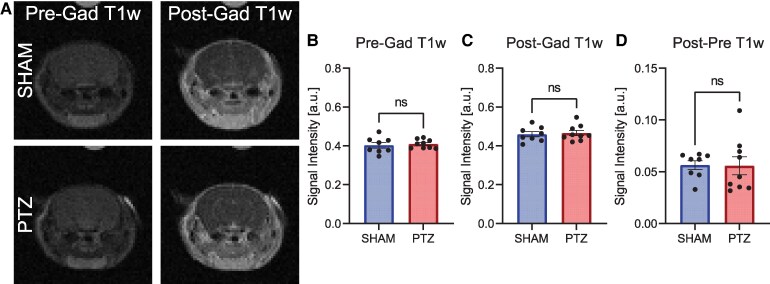
**Gadolinium administration demonstrates minimal difference in BBB permeability after PTZ kindling.** (**A**) T1w MRI images taken pre- and post-gadolinium administration in SHAM and PTZ mice. T1w MRI intensities from ROIs in the ventral hippocampus and temporal cortices before injection of a gadolinium-based contrast agent (**B**) (*P* = 0.6822, *n* = 8 SHAM, *n* = 9 PTZ mice, *t* = 0.4175, two-tailed unpaired *t*-test), after injection (**C**) (*P* = 0.7558, *n* = 8 SHAM, *n* = 9 PTZ mice, *t* = 0.3167, two-tailed unpaired *t*-test), and their respective difference (**D**) (*P* = 0.9523, *n* = 8 SHAM, *n* = 9 PTZ mice, t = 0.061, two-tailed unpaired *t*-test) demonstrate no difference in BBB integrity between SHAM and PTZ mice. ns: *P* > 0.05 (non-significant).

One significant potential confounding factor in this study was the integrity of the animal’s BBB, as PTZ kindling may disrupt its function,^54,55^ and the transport of Pyr across the BBB following systemic administration is a rate-limiting step in the conversion of ^13^C HP Pyr to Lac in brain. To assess BBB integrity, we used gadolinium-enhanced T1w MRI following PTZ kindling (Fig. 3). Images before and after injection of the contrast agent from three representative animals in each of the two groups are shown in Supplementary Fig. 2. Our analysis showed no significant difference between PTZ and sham for the intensities in T1w MRI pregadolinium (*P* = 0.6822), post-gadolinium (*P* = 0.7558), or their respective difference (*P* = 0.9523) (Fig. 3, Supplementary Fig. 2). This suggests that the BBB was relatively unaffected, at least for large molecules such as G-macro complexes, at the time point used in our model and that ^13^C HP Pyr transport across the BBB likely was unaffected by PTZ treatments.

### HP ^13^C MRSI lactate measurements correlate with true lactate concentrations

We evaluated the accuracy of our HP ^13^C MRSI measurements in detecting relative Lac concentrations in the brain by conducting a correlation analysis between our *in vivo* MRSI of HP [1-^13^C]Pyr measurements and Lac assay measurements from harvested brains of sham and PTZ-kindled mice. We found a correlation between these two variables (*r* = 0.6559, *R*^2^ = 0.4302, *P* = 0.0042), indicating that our HP ^13^C MRSI successfully detected true biological elevations in Lac production induced by PTZ kindling (Fig. 2E).

## Discussion

In this study, we demonstrate that MRS/I of HP [1-^13^C]Pyr can successfully detect differences in Lac production in *in vitro* and *in vivo* models of epilepsy. Several prior studies have shown the effectiveness of this technique in characterizing Pyr conversion to metabolites such as Lac *in vivo*, particularly in conditions like glioma^62^ and traumatic brain injury.^63^ This is the first study to assess whether HP ^13^C MRSI can differentiate healthy from highly active cultured neurons and epileptic brain tissue by detecting elevated Lac production.

Using two different epilepsy models, we showed that HP ^13^C MRS/I was able to distinguish normal (control) from epileptic conditions after chronic seizure activity at both cellular and tissue levels. First, we demonstrated that HP ^13^C MRS successfully identified elevated Lac/Pyr ratios in low Mg^2+^ treated cells compared to untreated controls. This finding aligned with elevated Lac levels measured by Lac assay in low Mg^2+^ treated cells (Fig. 1). Second, we showed *in vivo* that HP ^13^C MRSI successfully identified elevated Lac/Pyr ratios in epileptic mice within specific brain regions associated with seizure generation induced by PTZ kindling.^59-61^ As observed in the *in vitro* experiments, this result was further supported by elevated brain Lac concentrations measured independently by a Lac assay in PTZ-treated animals (Fig. 2). The moderate correlation between HP ^13^C MRSI Lac/Pyr ratios and Lac assay measurements across individual animals (Fig. 2E) validated the accuracy of our technique in detecting true biological increases in Lac production. Taken together, these results suggest that MRSI of HP [1-^13^C]Pyr can effectively detect increased Lac production resulting from epileptic neuronal activity.

While our results are promising, we acknowledge some limitations. For *in vitro* experiments, HP ^13^C MRS measurements were performed on live cells actively converting Pyr to Lac, whereas Lac assay measurements were done on supernatants of cells collected just before scanning. This means that HP ^13^C MRS detected intracellular Lac production (i.e. LDH activity), while Lac assay measurements detected extracellular Lac (i.e. the chemical pool of Lac) produced before the HP ^13^C MRS scans. The reported values for Lac/Pyr assume that the Pyr and Lac signals come from the same distribution. It is possible that after the injection of the Pyr solution cells precipitate/accumulate at the bottom of the syringe where the local RF excitation and sensitivity profile is lower. As the Lac signal originates only from the cells this would lead to an underestimation of Lac/Pyr. Considering the size of the RF surface coil and the volume of the solution we estimate that Lac/Pyr would be underestimated by approximately 20% for the extreme case that all cells would be at the bottom of the syringe while all the Pyr signal would be in the region of highest sensitivity. However, even if the precipitation is different for Mg^2+^ treated and control cells it would not explain the almost 3-fold higher Lac/Pyr measured in Mg^2+^ treated cells. When comparing the relative signal intensities for *in vitro* and *in vivo* MRS/I experiments, the relatively low Lac signal amplitude (∼1/1000 of the Pyr peak) is predominantly due to the high Pyr concentration (∼7 mM) relative to the cell density (∼1 × 10^7^ cells in 4 mL solution) that converts Pyr into Lac. This is consistent with previous reports on HP ^13^C studies under similar experimental conditions.^64^

For *in vivo* experiments, HP ^13^C MRSI signals were measured in specific ROIs in the temporal cortex/hippocampus to remove confounding vascular structures, whereas Lac assay measurements were done with whole hemisphere homogenates to ensure sufficient sample amounts were obtained while minimizing tissue processing time for proper Lac concentration measurements. Further, while we performed all brain harvesting procedures, including perfusion and extraction in ice-cold conditions, it is possible that rapid Lac concentration changes post-mortem^65^ could have affected measured Lac and contributed to differences between HP ^13^C MRSI and Lac assay results. Additionally, we only used female mice to account for intersex differences in PTZ seizure susceptibility, ensuring all animals experienced similar seizure burdens.^66-68^ This approach may have overlooked potential sex-specific effects on the efficacy of MRSI of HP [1-^13^C]Pyr in detecting epileptic brain tissue. Lastly, while we aimed at placing the RF surface coil centred with respect to the animal’s brain, there may have been variability in the exact positioning. Surface coils offer high signal-to-noise ratio near the surface but less homogeneous field profiles than volume coils, limiting use for larger areas. Variability in the distance between the coil and the skull surface could affect overall signal intensity as well as signal-to-noise ratio. While the Lac/Pyr ratio is insensitive to inhomogeneities in the RF receive profile, differences in the RF transmit profile will affect this metric. Considering small nominal flip angles of the used multi-band spectral-spatial RF pulse, a ±20% deviation in RF transmit power translates into a similar deviation in effective flip angles that would lead to an approximately ±10% deviation in Lac/Pyr. For comparison, using only a spatially selective RF pulse with 2° excitation for both Pyr and Lac, the same deviation in RF power would lead to a deviation in Lac/Pyr of less than 3%. However, this would be at the cost of a ∼50% reduction in Lac signal-to-noise ratio. Using a volume RF with a more homogeneous transmit profile in combination with a surface receive-only coil would ameliorate this issue. A dual-tuned ^1^H/^13^C volume RF coil for mouse brain imaging would also provide higher quality ^1^H MRI data. The use of a ^1^H volume coil with a large diameter (72 mm) here enabled sequential ^1^H and ^13^C imaging without repositioning the animal. However, this setup contributed to the relatively low quality of the anatomical proton images.

These findings align with extensive literature suggesting that non-invasive imaging of Lac and other metabolites such as glutamate, GABA and glucose holds significant promise for improving brain mapping of seizure onset zone and epileptic networks for resection surgery.^10-12,25-31,69^ While previous Lac imaging studies in epilepsy focused on using standard ^1^H MRS, our findings demonstrate that HP ^13^C MRSI, with potentially improved spatial resolution, can effectively delineate epileptic regions in brain tissue. Precise delineation of tissues involved in the seizure onset zone and epileptic network prior to resection is critical for maximizing post-resection seizure freedom.^70^ Therefore, MRSI of HP [1-^13^C]Pyr may be a superior tool compared to conventional MRS for mapping the seizure onset zone and epileptic network. Future studies should directly compare the sensitivity and accuracy of Lac detection in epileptic tissue using conventional ^1^H versus HP ^13^C MRSI. Additionally, we intend to explore the use of HP ^13^C MRS/I preclinically with resected human tissue samples and eventually in clinical trials to assist in brain mapping of epileptic regions. Differential LDHA expression and elevated lactate production have been previously detected in epilepsy patients in approximately 1 cm^3^ brain tissue volume, well within the spatial resolution limits of clinical HP ^13^C MRSI techniques.^13,15,23,62,71,72^ Once the effectiveness of HP ^13^C MRSI-assisted resection mapping is established in patients, it will be important to elucidate variability in Lac signals detected in sub-types of epilepsy, such as complex partial versus non-convulsive generalized seizures.

Overall, our study successfully demonstrated a novel application of MRS/I of HP [1-^13^C]Pyr in *in vitro* and *in vivo* models identifying Lac production as a key biomarker of epileptic brain activity. We anticipate that this study will provide crucial preclinical evidence to support further exploration in epilepsy patients.

## Conclusions

In this study, we validate the reliability of MRS/I of HP [1-^13^C]Pyr in detecting differences in Lac production between control and epileptic neural tissues *in vitro* and in a rodent model of epilepsy. This demonstrates the potential for HP ^13^C MRSI to serve as a viable tool for non-invasive mapping of epileptic brain based on metabolic activity. These findings pave the way for improved identification of the seizure onset zone and nodes within the epileptic network, which are crucial for guiding epilepsy surgery. We believe these data will facilitate the clinical use of HP ^13^C MRSI in assisting with epilepsy surgeries, ultimately enhancing post-resection seizure freedom outcomes. Future studies, including preclinical trials using resected human tissue samples and eventual clinical trials, will be essential to establish the effectiveness and utility of HP ^13^C MRSI in clinical practice.

## Supplementary Material

fcaf357_Supplementary_Data

## Data Availability

The authors confirm that the data supporting the findings of this study are available within the article, in Supplementary material, and the OSF repository DOI 10.17605/OSF.IO/BP4DH (https://osf.io/bp4dh/) together with the code generated for this study.
