# Supplementary material for: Hyperpolarized [1-13C]pyruvate magnetic resonance spectroscopic imaging identifies elevated lactate in epileptic tissue
Source: Brain Commun. 2025 Sep 13;7(5):fcaf357. doi: 10.1093/braincomms/fcaf357 (PMC12464677; doi:10.1093/braincomms/fcaf357)
Supplement: fcaf357_Supplementary_Data [file fcaf357_supplementary_data.pdf]

Supplementary Materials

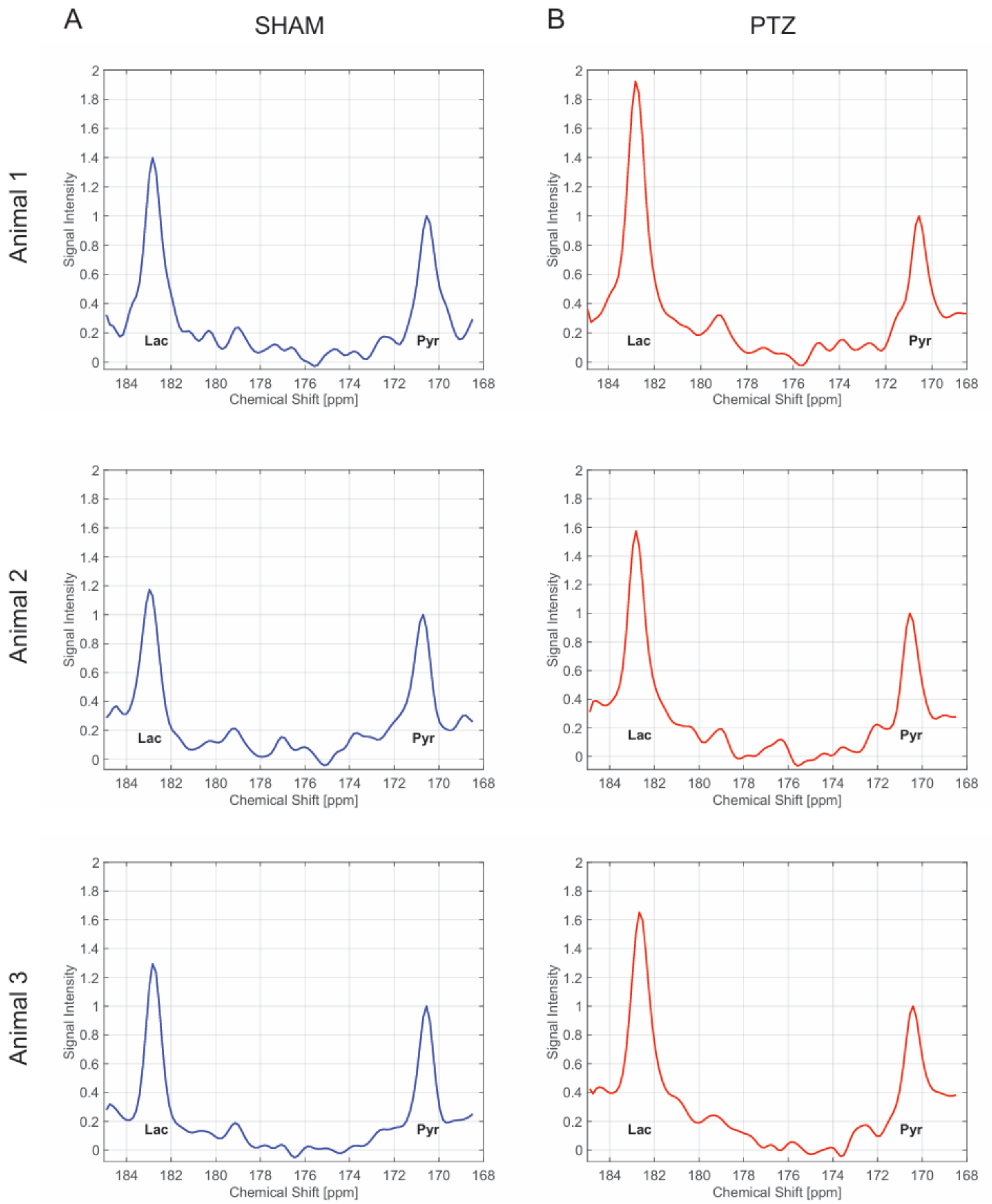

Supplementary Figure 1: Representative Spectra for In Vivo HP 13C MRS Experiments. HP ^{13}C MRS spectra normalized to maximum Pyr from ROIs in the ventral hippocampus and temporal cortices from three representative SHAM (A) and PTZ (B) animals.

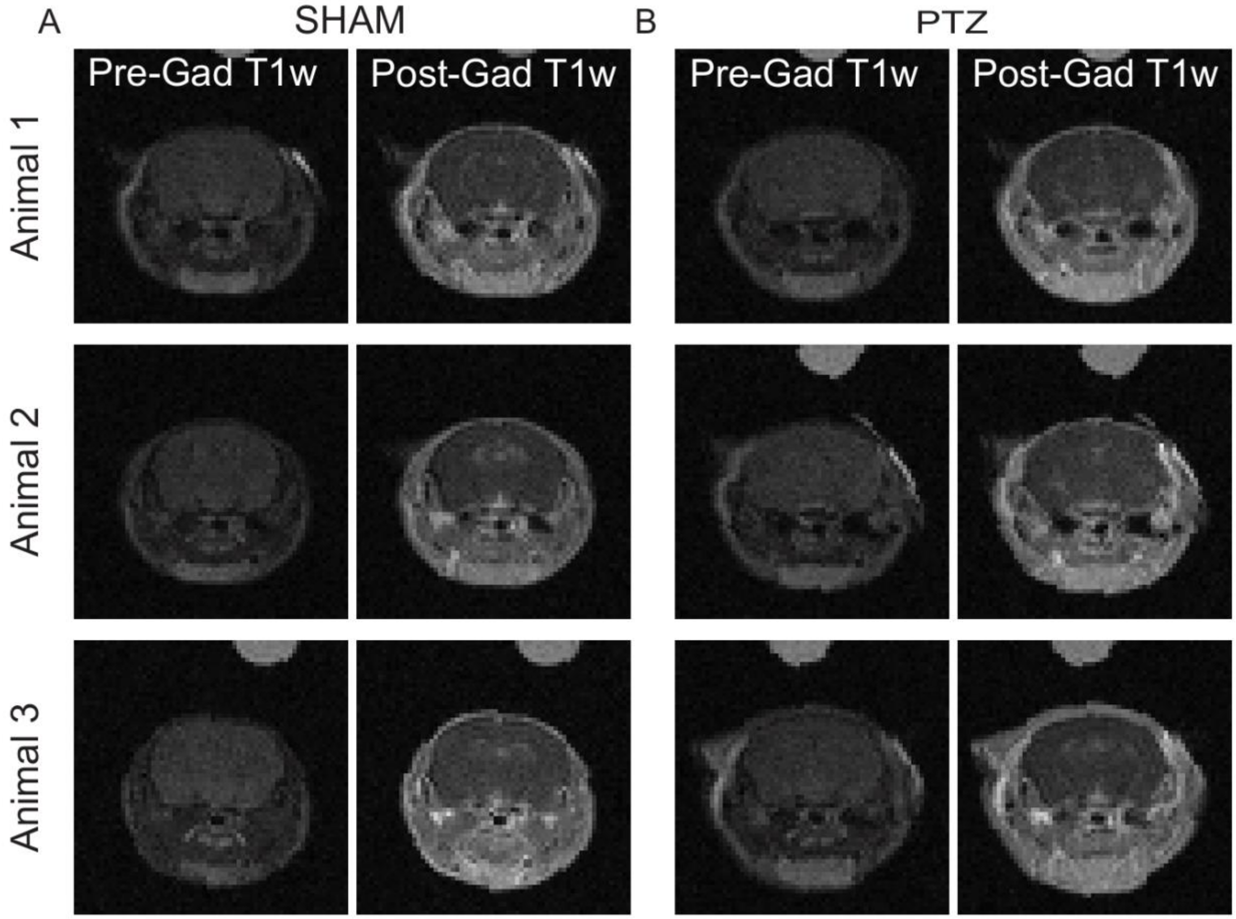

Supplementary Figure 2: Representative MRI Images Pre and Post-Gadolinium. T1W MRI before and after injection of a gadolinium-based contrast agent from three representative SHAM (A) and PTZ (B) animals.
